# The Effect of Created Local Hyperosmotic Microenvironment in Microcapsule for the Growth and Metabolism of Osmotolerant Yeast *Candida krusei*

**DOI:** 10.1155/2013/467263

**Published:** 2013-11-05

**Authors:** Guo Chen, Shanjing Yao

**Affiliations:** ^1^Department of Biotechnology and Bioengineering, Huaqiao University, Xiamen 361021, China; ^2^Department of Chemical and Biochemical Engineering, Zhejiang University, Hangzhou 310027, China

## Abstract

*Candida krusei* is osmotolerant yeast used for the production of glycerol. Addition of osmolyte such as NaCl into culture medium can increase the production of glycerol from glucose, but osmolytes may burden the glycerol separation. A coencapsulation method was suggested to create local extracellular hyperosmotic stress for glycerol accumulation. Firstly, the influence of osmotic stress induced by the addition of PEG4000 on growth and metabolism of free cell was studied in detail. Glycerol accumulation could be improved by employing PEG4000 as osmoregulator. Secondly, cells and PEG4000 were coentrapped in NaCS/PDMDAAC capsules to create local hyperosmotic stress. The effects of local hyperosmotic microenvironment on the cell growth and metabolism were studied. The coentrapment method increased the glycerol concentration by 25%, and the glycerol concentration attained 50 gL^−1^ with productivity of 18.8 gL^−1^Day^−1^ in shake flask. More importantly, the glycerol could be directly separated from the encapsulated cells. The entrapped cells containing PEG4000 were also cultivated for 15 days in an airlift reactor. The yield and productivity were ca. 35% and 21 gL^−1^Day^−1^, respectively.

## 1. Introduction

Glycerol is an important chemical used in many fields. The fermentation process for production of glycerol was firstly commercialized in Germany during World War I. The yeasts usually used for production of glycerol were *Saccharomyces cerevisiae*, *Pichia farinose*, *Candida glycerinogenes*, and *Candia krusei *[1]. Recently, glycerol production through osmotolerant yeasts, such as *P. farinos, C. glycerinogenes*, and *C. krusei*, received more attention [2–4]. Generally, osmotolerant yeast synthesizes glycerol as a compatible substance to balance the extracellular hyperosmotic stress. Therefore hyperosmotic stress can incur osmotolerant yeasts to produce more glycerol. Glycerol 3-phosphate dehydrogenase is the key enzyme for glycerol production. Heterologous expression of Glycerol 3-phosphate dehydrogenase gene [*DhGPD1*] from *Debaryomyces hansenii* in *Saccharomyces cerevisiae* showed that the gene was functional in *S. cerevisiae*, but its heterologous expression was not efficient to osmotic stimulation, which suggested that the regulatory mechanism may not be shared by these two yeasts [5, 6]. The effects of hyperosmotic stress on osmotolerant yeasts *Zygosaccharomyces rouxii* and *Pichia sorbitophila* and the less osmotolerant *Saccharomyces cerevisiae* were investigated by Djelal et al. [7]. The results demonstrated that osmolytes were not metabolized but mainly released upon exposure to hypotonic conditions. Kayingo et al. [8] studied the effect of medium osmolality on the production of glycerol and ethanol by *Hansenula anomala* growing on glucose and ammonium. The study on the mechanisms of glycerol accumulation showed that maintaining high extracellular osmotic pressure is necessary for glycerol accumulation by osmotolerant yeast. Addition of osmoregulators such as carbohydrates and inorganic salts into medium was proved to be effective in stimulating the production of glycerol [9, 10]. However, the addition of osmoregulators would burden the separation of downstream.

Immobilization was efficient for the cell cultivation and fermentation with advantages, such as high cell density, prevention of washout, high yield, and low risk of contamination [11]. Report about immobilized osmotolerant yeasts for the production of glycerol has appeared in the literature [12]. In free cultivation, the high concentration of glucose was usually used to provide initial osmotic environment for osmophilic yeasts. Wherever cells were immobilized in beads or capsules, the osmotic stress in microenvironment around the yeast was not high enough to induce the high activity of glycerol-3-phosphate dehydrogenase (GPDH) which was key enzyme for high production of glycerol [13]. This may be caused by diffusion rate limitation of substrate into beads.

In this work, a new method was developed to create local hyperosmotic stress around the osmotolerant yeast for the production of glycerol. The capsule prepared from sodium cellulose sulfate (NaCS) and polydimethyl-diallyl-ammonia chloride (PDMDAAC) was used to coencapsulate the osmotolerant yeast *Candida krusei* and PEG4000 for the production of glycerol. The time evolution of cells, glucose, and glycerol concentration for free cell and encapsulated cell with addition of PEG4000 was analyzed.

## 2. Materials and Methods

### 2.1. Materials

Polydimethyl-diallyl-ammonia chloride (PDMDAAC, MW 200,000–350,000) was obtained from Aldrich (Germany). Sodium cellulose sulfate (NaCS) was prepared by heterogeneous reaction as described before in our lab [14]. Polyethylene glycol (PEG) with different average molecular weight such as PEG500, PEG1500, PEG2000, PEG4000 and PEG6000 was purchased from Sangon (Shanghai, China). All other reagents for preparation of culture medium were of analytical reagent grade and were purchased from local suppliers.

### 2.2. Microorganism and Medium

In all experiments, the strain used was osmotolerant yeast *Candida krusei* (ICM-Y-05) obtained from the Institute of Process Engineering, Chinese Academy of Sciences Beijing, China. The agar slant medium contained 100 g L^−1^ glucose, 3 g L^−1^ urea, 3 g L^−1^ corn steep liquor, and 20 g L^−1^ agar. For seed cultures, the medium contained 100 g L^−1^ glucose, 3 g L^−1^ urea, and 3 g L^−1^ corn steep liquor. For fermentation, the medium composition was 200 g L^−1^ glucose, 2.5 g L^−1^ urea, 3 g L^−1^ corn steep liquor and 3.5 g L^−1^ KH_2_PO_4_. 

### 2.3. Preparation of Capsules

Cell entrapment in the capsules was carried out under sterile condition as follows. Sterilized water of 200 mL was added into 50 mL *C. krusei* seeds to obtain diluted cell solution. Diluted cell solution of 250 mL was mixed with 8.75 g NaCS and stirred to form homogeneous solution. Then the solution was degassed and added drop wise by a syringe into 6% (*w*/*v*) PDMDAAC solution. Capsules were formed at room temperature under mild stirring for 40 mins. Then microcapsules were washed with sterilized water to eliminate excess free PDADMAC on the surface of capsule. To create the local hyperosmotic stress, PEG was dissolved in prepared NaCS solution, and PEG would be limited in capsules, while NaCS solution was dropped into PDMDAAC solution. And then capsules entrapped different PEG were placed into deionized water for 500 mins to determine the diffusion of PEG from inner capsule. Then appropriate volume of capsules was added into fermentation medium to carry out fermentation. 

### 2.4. Culture Condition

The seed slant was incubated at 35°C for 24 h and stored at 4°C. Seed was precultured aerobically in shaking flask containing 100 mL seed medium at 35°C on a rotary shaker (150 rpm) for 24 h. For fermentation, a volume of 5 mL precultured seeds or 25 mL capsules prepared as above was transferred into 500 mL shake-flask containing 50 mL fermentation medium and then followed by 5-day incubation at 35°C on a rotary shaker (150 rpm). For batches of fermentation, 150 mL capsules containing *C. krusei* seeds were added into 450 mL fermentation medium in an airlift loop reactor. The volume of capsules could be calculated by the volume difference between the final volume and initial medium volume.

### 2.5. Compression Intensity of Capsules

The compression intensity of the capsules was examined using an Electronic Universal Testing Machine (CSS-44001, Changchun Research Institute for Testing Machines, China) at room temperature with a compressing rate of 5 mm/min until the capsule ruptured. Twenty replicates were tested and averaged.

### 2.6. Diffusion Behavior of Glucose into Capsule

The capsules were taken out at a different culture time interval and washed three times with deionized water. Then the capsules were submerged in 0.9% NaCl solution until the glucose in bulk could not be tested. Then diffusion of glucose from the well-stirred solution into capsules was measured. Firstly same volume of capsules was added into well-stirred glucose solution at a temperature of 25°C, and samples of 50 *μ*L were collected at a defined time interval. Transfer coefficients were calculated from kinetic curves of glucose diffusion into capsules according to the following model [15]:

(1) $$\begin{array}{c} \frac{C}{C_{0}}=\frac{C_{\text{eq}}}{C_{0}}+\left(1-\frac{C_{\text{eq}}}{C_{0}}\right)\exp\left[-\left(\frac{1}{V_{0}}+\frac{1}{V_{C}}\right)kAt\right], \end{array}$$

where *C* is instant concentration of the bulk solution, *C*_eq_ is the concentration of bulk solution in equilibrium state, *C*_0_ is the initial concentration of the bulk solution, *V*_0_ is the volume of solution, *V*_*c*_ is the volume of capsules, *D* is the diameter of capsule, *k* is the mass transfer coefficient, *t* is time, and *A* is defined by 6 *V*_*c*_/*D*. In this model, the capsule was postulated to be homogeneous. Under perfect stirring, the liquid film resistance around the capsules could be ignored. 

### 2.7. Measurement of Biomass, Glucose, and Glycerol in Capsules and Broth

The dynamic profiles of cell, substrate and metabolites size were monitored during fermentation. Multiple flasks were run at the same time, and three flasks were sacrificed at each sampling point. Each datum point was expressed by an average with an error bar (i.e., standard deviation from three independent samples). Biomass concentration was estimated from the absorbance of appropriately diluted culture medium at 600 nm (Ultrospec3300 proUV/Visible) according to the predetermined correlation between optical density and dry weight of biomass. For the concentration of biomass in capsule, the capsule was broken by pressing, and the biomass was released to a definite volume of water. And then the biomass solution was drawn and the capsule fragment was removed. The biomass solution drawn was measured at 600 nm to determine the concentration of biomass in capsule. Glucose concentration was determined by the dinitrosalicylic acid (DNS) method [16]. Glycerol concentration was determined by periodate-chromotropic acid analysis [17]. The concentration of glucose and glycerol in broth was measured directly, while the concentration of biomass, glucose, and glycerol in capsules was measured after rupture of capsules.

Relative gross concentration (RGC) was a relative concentration calculated by (2) and (3) by assuming that the concentration of glucose and glycerol was zero inside capsule. Consider

(2) $$\begin{array}{c} C_{g(\text{glu})}=\frac{\left(C_{m(\text{glu})}\times V_{m}+C_{c(\text{glu})}\times V_{c}\right)}{V_{m}}, \end{array}$$

(3) $$\begin{array}{c} C_{g(\text{gly})}=\frac{\left(C_{m(\text{gly})}\times V_{m}+C_{c(\text{gly})}\times V_{c}\right)}{V_{m}}, \end{array}$$

(4) $$\begin{array}{c} \text{Yield}=\frac{C_{g(\text{gly})}}{\left(C_{i(\text{glu})}-C_{g(\text{glu})}\right)}, \end{array}$$

(5) $$\begin{array}{c} \text{Production}\;\text{rate}=\frac{\left(C_{g(\text{gly})}\right)}{t}, \end{array}$$

where *C*_*g*(glu)_, *C*_*m*(glu)_, and *C*_*c*(glu)_ are the RGC of glucose and the glucose concentration in broth and in capsule, respectively. *V*_*m*_ and *V*_*c*_ are the medium volume and capsule volume, respectively. *C*_*g*(gly)_, *C*_*m*(gly)_, *C*_*i*(glu)_, and *C*_*c*(gly)_ are RGC of glycerol, glycerol concentration in broth, the initial glucose concentration in broth, and glycerol concentration in capsule, respectively. *t* is the time of fermentation.

## 3. Results and Discussion

### 3.1. Effect of PEG4000 as Osmoregulator on Glycerol Production

**Figure 1:**
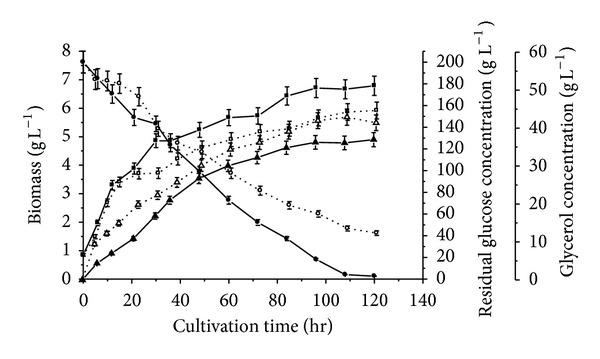
Time profiles of cell growth (□, ■), residual sugar level (○, ●), and glycerol concentration (△, ▲) by free cell of *C. krusei* at 35C. Open symbols: 2% (*w*/*v*) PEG4000 added into medium; dark symbols: no PEG4000 added into medium.

In the following research, PEG4000 was chosen as osmoregulator and entrapped in capsule to provide hyperosmotic stress. The effect of PEG4000 on cell growth and metabolism was investigated in Figure 1. Dash lines in Figure 1 described the effect of 2% (*w*/*v*) PEG4000 in medium on cell growth, glucose consumption, and glycerol production. The cell growth was inhibited by the PEG4000 added, which was in accordance with the results under high initial glucose concentration or addition of NaCl reported by Liu et al. [10] and Blomberg and Adler [18]. The final biomass concentration was 5.9 ± 0.3 g L^−1^ in the PEG4000-containing medium, compared with 6.8 ± 0.3 g L^−1^ in medium without PEG4000. The glucose was consumed more slowly in PEG4000-containing medium than that without PEG4000. And higher residual glucose concentration existed in PEG4000-containing medium. The highest glycerol concentration in process was 42.9 ± 2.1 g L^−1^ and 37.0 ± 1.8 g L^−1^ in the medium with PEG4000 and without PEG4000, respectively. The glycerol production was synchronous with cell growth. The results showed that the PEG4000 was similar to NaCl as osmolyte on cell growth and glycerol production. 

### 3.2. Creation of Local Hyperosmotic Stress for Glycerol Production

#### 3.2.1. Selection of PEG

Due to the mass transfer limitation, the high concentration of glucose in medium could not provide extracellular hyperosmotic stress for immobilized cell. If the glucose concentration in medium was too high, the capsules would shrink sharply and could not return to its original shape even after a long time. As we know, the substances with high molecular weight could not permeate through NaCS-PDMDAAC membrane of capsule [19]. If cutoff substances were entrapped in capsules, local hyperosmotic pressure environment inside capsules could be created in long duration. In this work, several kinds of PEG with different average molecular weight (MW) were selected as high molecular matter to entrap in capsule. 

**Figure 2:**
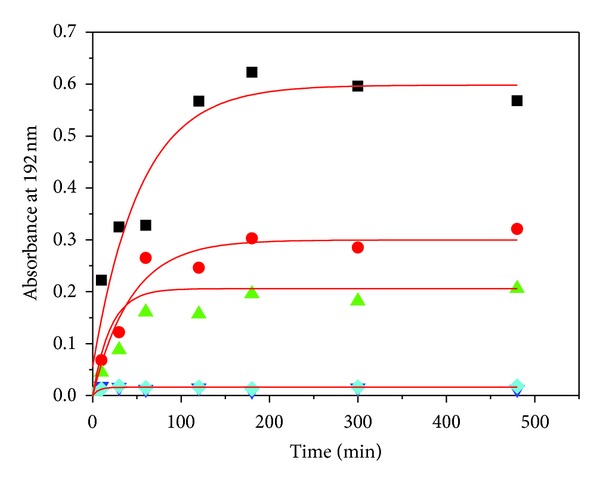
Kinetic curves of PEG with different average molecular weight diffusion out of capsules. Symbols in figure: PEG500 (■), PEG1500 (●), PEG2000 (▲), PEG4000 (*▼*), and PEG6000 (◆).

**Figure 3:**
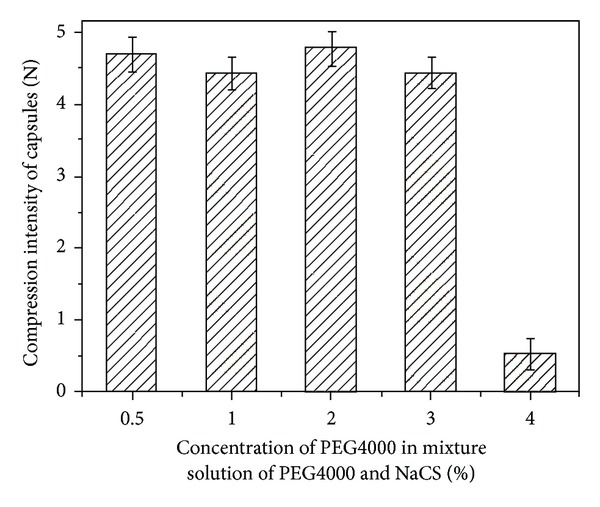
Effect of different PEG4000 concentration on mechanical strength of capsules.

The diffusion experiments for PEGs with different MW entrapped in capsule determined the molecular weight of PEG used in the following experiment. The results in Figure 2 showed that PEG2000 can permeate through the membrane of NaCS-PDMDAAC capsules to solution, and PEGs with MW larger than 4000 hardly could permeate out of capsules. So PEGs with MW above 4000 were selected as osmoregulators entrapped in capsule to create local hyperosmotic stress. The effect of different PEG4000 concentration on mechanical strength of capsule during the capsule formation was studied in Figure 3. If the concentration of PEG4000 was less than 3% (*w*/*v*), the mechanical strength of capsules kept quite well.

**Figure 4:**
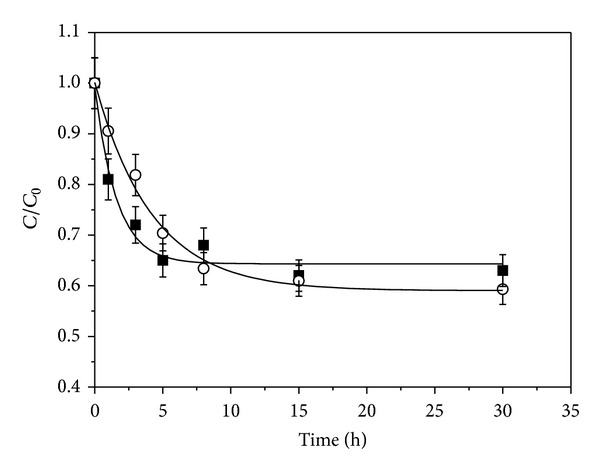
Comparison of glucose diffusion into capsules containing PEG4000 (○) with those capsules without PEG4000 (■).

The kinetic curves of glucose diffusion into capsules with PEG4000 and without PEG4000 were compared in Figure 4. By calculating with (1), the glucose transfer coefficients were 0.056 mm/min and 0.14 mm/min in capsules with PEG4000 and without PEG4000, respectively.

#### 3.2.2. Culture in Shake Flask

**Figure 5:**
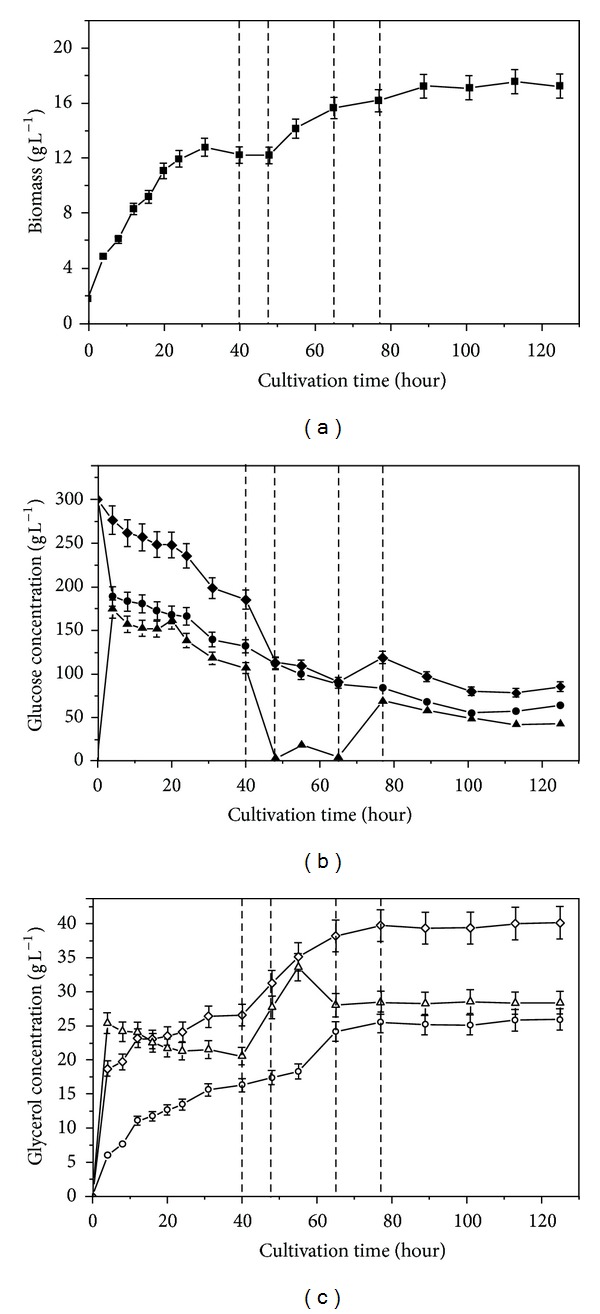
Time course of biomass (■), glucose concentration (closed symbol), and glycerol concentration (open symbol) in capsule (▲, △) and medium (●, ○) for encapsulated *C. krusei* without PEG4000 (CWOP). Relative gross glucose concentration (◆) and relative gross glycerol concentration (*◊*).

**Figure 6:**
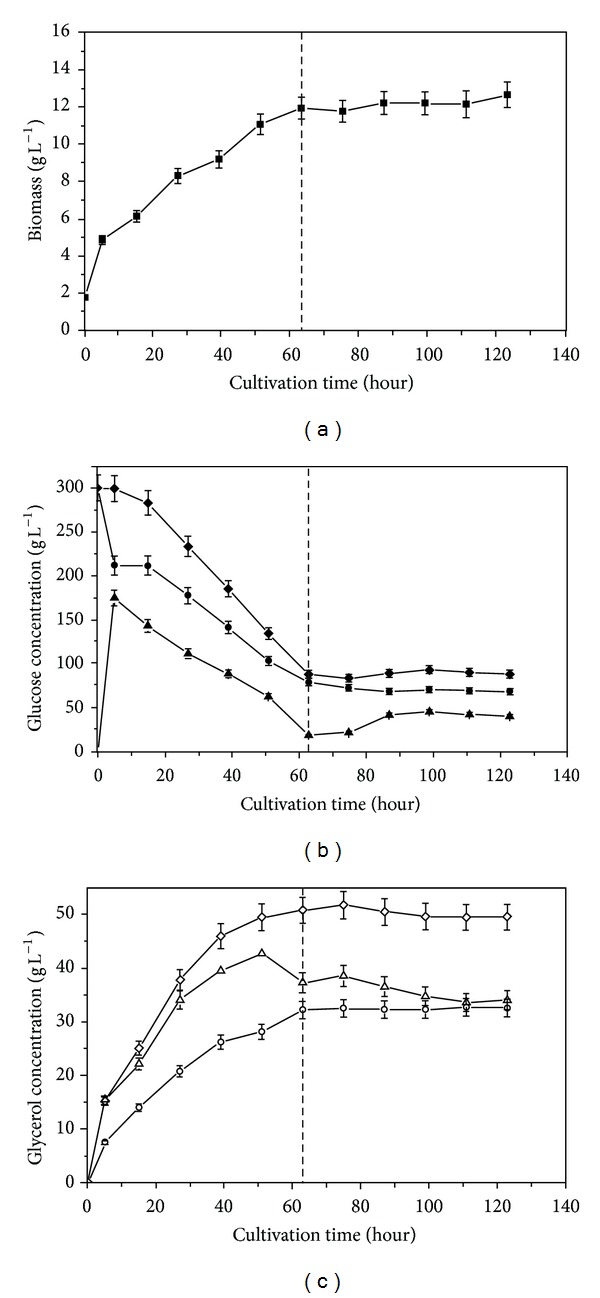
Time course of biomass (■), glucose concentration (closed symbol), and glycerol concentration (open symbol) in capsule (▲, △) and medium (●, ○) for encapsulated *C. krusei* with 3% (*w*/*v*) PEG4000 (CWP). Relative gross glucose concentration (◆) and relative gross glycerol concentration (*◊*).

The capsules with PEG4000 and without PEG4000 were abbreviated as CWP and CWOP.  Figure 5 showed the time evolution of cell growth, glucose, and glycerol concentration in medium and capsules when cells in CWOP were fermented. From Figure 6(a), the cell began stationary phase at ca. 64 h. And the maximal cell concentration reached 12 g L^−1^ which was lower than 16 g L^−1^ in CWOP as Figure 5. The inhibition phenomenon of PEG4000 was similar to free culture in medium in Figure 1. Due to the PEG4000 entrapped in capsules, the transfer coefficient of substrate diffusion into capsule was changed. The variation trend of glucose concentration in capsule was different from CWOP. Two stages of growth could not be observed in Figure 6, which was different from cell in CWOP as Figure 5. The consumption rate of glucose was relative uniform in Figure 6(b), not as abruptly decreasing at a certain time in CWOP. The consumption of glucose was also slower in CWP than in CWOP, because the cell grew more slowly in CWP than in CWOP. Final *C*_*g*(glu)_ was ca. 88 ± 4.4 g L^−1^ due to the osmotic balance in extra- and intracellular in 64 h. From Figure 6(c), the rate of glycerol production was faster than in CWOP as Figure 5(c). Final *C*_*g*(gly)_ was ca. 50 ± 2.5 g L^−1^ which was ca. 40 ± 2.1 g L^−1^ in Figure 5(c).  Table 1 showed comparison of final biomass, glucose concentration, glycerol concentration, yield and production by free and encapsulated cells. The results in Figure 6 showed that the hyperosmotic stress in capsule caused by PEG4000 could increase the glycerol accumulation by *C. krusei*. Therefore, the high molecular entrapped in capsules was an effective way to create local hyperosmotic stress.

### 3.3. Immobilized Cell Cultured in Airlift Reactor

The entrapped cells containing PEG4000 were cultivated for 15 days in five batches, and each batch lasted for three days. All the experiments were carried out in an airlift reactor. Results were shown in Table 2. From the third to fifth batch, the operation of the reactor was almost stable. And the yield and productivity were ca. 35% and 21 g L^−1^ Day^−1^, respectively. According to our observation, the medium was almost completely clear from first batch to fourth batch, which meant that leakage of cells from capsules were scarce during cycling cultivation. But the medium began to turn to turbidity in the fifth batch, which may be caused by some broken capsules. The oxygen transfer could be another issue for encapsulation technique used in glycerol production by fermentation. Here, an airlift reactor was used to provide enough oxygen during fermentation. In his small airlift reactor, the inhibition of oxygen transfer was not obviously observed in process. If this process was scaled up, the oxygen transfer needs strengthening to ensure the oxygen requirement of yeast. The encapsulation of cells can be used to carry out continuous fermentation. In the process, the medium after fermentation was easy to separate from the cells in capsules.

## 4. Conclusion

The results for production of glycerol using osmotolerant yeast of *C. krusei* indicated that the addition of PEG4000 would inhibit the cell growth and improve the glycerol production in free culture. The final cell concentrations were 5.9 g L^−1^ and 6.8 g L^−1^ for medium with PEG4000 and without PEG4000, respectively. Encapsulation of cells was an easy method to carry out continuous cultivation and ease the separation of glycerol from medium. PEG4000 was encapsulated together with *C. krusei* to provide a more adaptable microenvironment which was affiliated to the production of glycerol. This method could increase the glycerol concentration by 25%, compared with cell encapsulation without PEG4000. The glycerol concentration attained 50 g L^−1^, and productivity was 18.8 g L^−1^ Day^−1^ in shake flask. More important was that this method could reduce the separation trouble caused by addition of osmoregulators. And this result was also reproduced in airlift reactor for 5 batches. So capsules could be used to create local microenvironment adaptable to cell growth and fine chemicals production.

## Figures and Tables

**Table 1:** Comparison of final biomass, glucose concentration, glycerol concentration, yield, and production by free and encapsulated cells.

	Biomass (g L^−1^)	Residual glucose conc. (g L^−1^)	Glycerol conc. (g L^−1^)	Yield (%)	Productivity (g L^−1^ Day^−1^)
Free cell culture	6.8	2.7	37.0	18.8	10.2
Free cell culture with PEG4000	5.9	42.4	42.9	27.2	11.3
Encapsulated cell without PEG4000	16	85	40.0	18.6	12.6
Encapsulated cell with PEG4000	12	88	50.0	23.6	18.8

**Table 2:** Five batches of encapsulated cells cultivation in airlift reactor.

Batch	Initial glucose conc. (g L^−1^)	Biomass (g L^−1^)	Residual glucose conc. (g L^−1^)	Glycerol conc. (g L^−1^)	Yield (%)	Productivity (g L^−1^ Day^−1^)
1	300	12.8	65.8	47.2	20.2	15.7
2	200	16.5	23.6	52.7	29.8	17.6
3	200	18.2	17.2	63.8	34.9	21.3
4	200	17.6	16.5	65.6	35.7	21.9
5	200	18.8	12.3	62.7	33.4	20.9
